# Verifications of Medorrhinum

**Published:** 1890-02

**Authors:** G. W. Sherbino

**Affiliations:** Abilene, Texas


### VERIFICATIONS OF MEDORRHINUM.

G. W. SHERBINO, M. D., ABILENE, TEXAS.

Mr. A., of lymphatic temperament, has been troubled with nasal pharyngeal catarrh for several years. I had given him several seemingly indicated remedies, with only very little benefit.

He complains of his head feeling too full of blood on first awakening; pain over the eyes and nose.

Eyes more or less bloodshot at all times; a yellow appearance of the conjunctivæ, eyes burn and feel hot. Snores when sleeping; always sleepy after dinner (Phos.). If he goes to sleep soon after eating, cannot bear to rouse up again; wants to be let alone; always ready to get up in the morning.

Drinks a great deal of water at night, not any through the day; never hungry, but eats well when once he gets started; cares nothing for dainties; does not like acids; craves stimulants after dinner; cannot drink stimulants before twelve o'clock; they make him nervous; often vomits after dinner, and sometimes after supper; vomited matters smell very sour; "always thirsty after vomiting."

Anus itches a great deal at night; throat feels as if full of phlegm, which causes him to constantly clear his throat; hawks up phlegm, but usually swallows it. I rarely see him spit on floor or ground.

Hawking and spitting worse when first waking in the morning, worse when lying in a recumbent position. If he wakens up at night it is as bad as first waking in the morning; expectorates clear, transparent mucus, which is very tenacious; often feels as if there was a small piece of phlegm which, if he could get it up, he could quit clearing his throat.

Urine often starts, then stops again (Con.).

Feet are too hot at night; wishes to uncover them. Lies uncovered when first lying down; feet do not burn in the daytime.

Dreams troubled, of some one wishing to kill him, or he is trying to kill some one.

Disposition restless; time passes too slowly (Arg.-n., Helonias, Lilium-tig.), and that every one is so very slow when they do anything for him; wants to be out-of-doors.

Usually of a cheerful disposition, but if blue he feels as if he had hurt some one's feelings, or that his own had been hurt; always has these symptoms when despondent, depressed.

Itching on the calves of the legs; eruption; scratches till the blood runs; worse at night (Psorin); eruption disappears in summer, but returns when the weather gets cold (Alum, Rhus-tox.); red pimples, small.

Itching in the nose, as if the hairs caused it.

Sighing respiration; when trying to clear his throat throws his head back, puts the trachea on stretch, and presses against it with the hand. Medorrhinum CM (F.) cured.

